# Investigation of the Mechanism and Chemistry Underlying Staphylococcus aureus*'* Ability to Inhibit Pseudomonas aeruginosa Growth *In Vitro*

**DOI:** 10.1128/jb.00174-22

**Published:** 2022-10-11

**Authors:** Lasse Kvich, Stephanie Crone, Mads H. Christensen, Rita Lima, Morten Alhede, Maria Alhede, Dan Staerk, Thomas Bjarnsholt

**Affiliations:** a Department of Immunology and Microbiology, University of Copenhagen, Copenhagen, Denmark; b Center for Surgical Science, Department of Surgery, Zealand University Hospital, Køge, Denmark; c Department of Drug Design and Pharmacology, University of Copenhagen, Copenhagen, Denmark; d Department of Clinical Microbiology, Copenhagen University Hospital Rigshospitalet, Copenhagen, Denmark; University of California San Francisco

**Keywords:** *Staphylococcus aureus*, *Pseudomonas aeruginosa*, interspecies interactions, multispecies infections, chronic infections, cystic fibrosis, wounds

## Abstract

Pseudomonas aeruginosa inhibits or eradicates Staphylococcus aureus in most in vitro settings. Nonetheless, P. aeruginosa and S. aureus are commonly isolated from chronically infected, nonhealing wounds and lungs of people with cystic fibrosis (CF). Therefore, we hypothesized that S. aureus could protect itself from P. aeruginosa through glucose-derived metabolites, such as small organic acids, preventing it from being eradicated. This *in vitro* study demonstrated that S. aureus populations, in the presence of glucose, secrete one or more substances that efficiently eradicate P. aeruginosa in a concentration-dependent manner. These substances had a molecular mass lower than three kDa, were hydrophilic, heat- and proteinase-resistant, and demonstrated a pH-dependent effect. Nuclear magnetic resonance analysis identified acetoin, acetic acid, and oligopeptides or cyclic peptides in glucose-grown S. aureus supernatants. All the tested wild-type and clinical S. aureus strain inhibited P. aeruginosa growth. Thus, we proposed a model in which a cocktail of these compounds, produced by established S. aureus populations in glucose presence, facilitated these two species' coexistence in chronic infections.

**IMPORTANCE** Chronic infections affect a growing part of the population and are associated with high societal and personal costs. Multiple bacterial species are often present in these infections, and multispecies infections are considered more severe than single-species infections. Staphylococcus aureus and Pseudomonas aeruginosa often coexist in chronic infections. However, the interactions between these two species and their coexistence in chronic infections are not fully understood. By exploring in vitro interactions, we found a novel S. aureus-mediated inhibition of P. aeruginosa, and we suggested a model of the coexistence of the two species in chronic infections. With this study, we enhanced our understanding of the pathogenesis of chronic multispecies infections, which is crucial to paving the way for developing improved treatment strategies.

## INTRODUCTION

Chronic bacterial infections established in nonhealing wounds and the lungs of patients with cystic fibrosis (CF) are significant and growing health concerns. CF is the most common hereditary disease in the Caucasian population, and chronic lung infection is the leading cause of mortality in this group of patients (1). Chronic wound infections affect approximately 1 to 1.5% of the population in the Western world, and this number is expected to increase over time (2). Aggregated bacteria, known as biofilms, are present in most chronic infections and these bacterial biofilms are difficult to eradicate as they become tolerant to antibiotics and are protected from the host defense system (3). Furthermore, chronic infections are often polymicrobial and increased pathogenicity, persistence, and antimicrobial tolerance in multispecies infections have been observed compared to single-species infections (4, 5). These results suggest that interspecies interactions may play a role in the severity of infections. However, the mechanisms of interspecies interactions are still not fully understood.

Pseudomonas aeruginosa and Staphylococcus aureus are among the most common bacteria causing chronic infections. They often dominate the infection and are found together in the lungs of CF patients and chronic wounds (6–12). S. aureus most often precedes P. aeruginosa colonization in CF lungs and chronic wounds, and later they co-exist (6, 9–11). However, we have previously shown that the two species do not mix in sputum from CF lungs or chronic wounds but are found in separated aggregates (7, 11, 13). Moreover, *in vitro* studies with *in vivo*-resembling models supports this spatial separation (14). Spatially separated bacterial species are valid for many chronic infections where they co-exist but are found in single-species aggregates, side by side, not as mixed-species aggregates (3, 15).

Although P. aeruginosa and S. aureus are known to coexist *in vivo*, most researchers have studied the inhibitory effect of P. aeruginosa. Several studies have described how P. aeruginosa quickly overgrows S. aureus *in vitro* (4, 16–19), and several P. aeruginosa substances that reduce the growth of S. aureus have been described, e.g., pyocyanin, LasA protease, hydrogen cyanide, and 4-hydroxy-2-alkylquinolines (HAQs), such as 4-hydroxy-2-heptylquinoline-N-oxide (HQNO) (12, 18, 20–25). However, because S. aureus was not overgrown by P. aeruginosa *in vivo*, we hypothesized that it could protect itself from P. aeruginosa, enabling *in vivo* coexistence. One mechanism whereby S. aureus could protect itself is through the breakdown of glucose, creating organic acids known to kill P. aeruginosa (26–28). Elevated glucose levels have been found in CF sputum, and CF patients with high blood glucose levels are more likely to be coinfected with both strains than patients with normal blood glucose levels (29, 30). Therefore, this study aimed to determine if S. aureus was protected from P. aeruginosa and if glucose played a role in the coexistence observed *in vivo*.

## RESULTS

### S. aureus acidified the growth medium when glucose was present and restricted the growth of P. aeruginosa.

**FIG 1:**
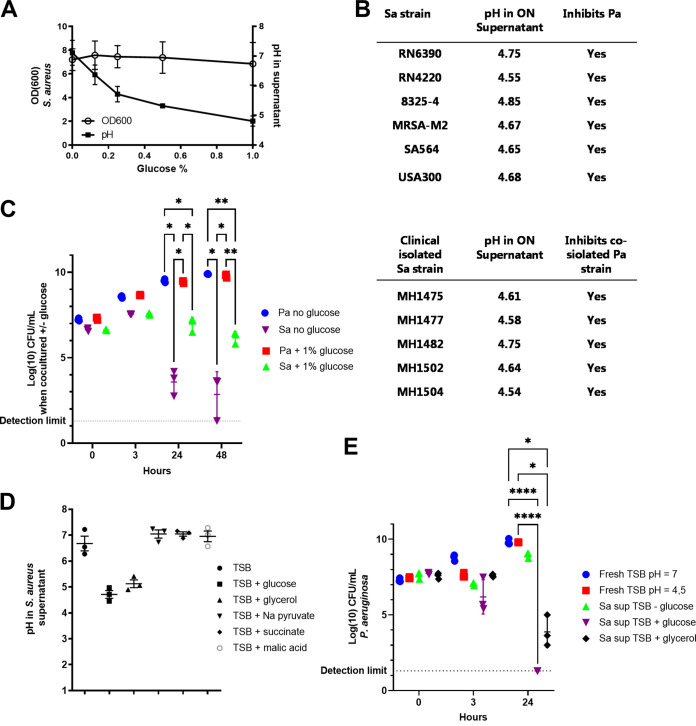
Staphylococcus aureus acidified the growth medium when glucose was present and restricted the growth of Pseudomonas aeruginosa. The relative growth (measured as absorbance at an optical density of 600 nm, OD_600_) and pH of S. aureus were measured in ON shaking cultures with 0 to 1% glucose (A). Glucose (1%) induced a low pH in various S. aureus supernatants, and the growth of P. aeruginosa in these supernatants was restricted (B). Simultaneous coculturing of S. aureus and P. aeruginosa, with or without 1% glucose, killed S. aureus over time (C). pH in S. aureus supernatants after growth with alternative carbon sources (D). The viability of P. aeruginosa (determined by log-transformed CFU per milliliter, log_10_ CFU/mL) was affected in S. aureus supernatants with either glucose or glycerol (E). Error bars represent standard deviation (SD). Statistical difference was calculated using a two-way ANOVA with Tukey's correction (*n* = 3, except [B] *n* = 1). *, adjusted *P*  < 0.05; **, adjusted *P* < 0.01; ****, adjusted *P* < 0.0001. Pa, P. aeruginosa; Sa, S. aureus; sup, supernatant; TSB, tryptic soy broth. Clinical S. aureus and co-isolated P. aeruginosa strains from patients with cystic fibrosis were collected from the Department of Clinical Microbiology at Rigshospitalet.

Glucose (0 to 1%) did not affect the growth of S. aureus shaking cultures, while pH decreased in a concentration-dependent manner (Fig. 1A). Similar results were observed for other S. aureus strains (clinical and laboratory strains, refer to Table S1 in Supplemental File 1), with pH values around 7 when no glucose was added to the growth medium (unpublished data) and pH values between 4.5 and 4.8 when 1% glucose was added (Fig. 1B). When grown in medium containing 1% glucose, all tested S. aureus supernatants displayed a P. aeruginosa inhibiting feature (Fig. 1B). In addition, S. aureus strains isolated from CF-lungs inhibited co-isolated P. aeruginosa strains (Fig. 1B). This inhibitory effect was not due to the change in pH, as shown below.

These data contrasted with what was observed when cocultured simultaneously in a 1:1 ratio in shake flasks. Here, P. aeruginosa outgrew S. aureus, similar to previously reported findings (16, 18, 31, 32). After 24 h, a significant reduction of S. aureus cells was detected in cultures with or without 1% glucose compared to P. aeruginosa. However, a reduced killing of S. aureus was observed in cultures with glucose (Fig. 1C). A log difference of 3.38 (SD = 0.78; *P* = 0.02) was found between S. aureus cultures with and without glucose after 24 h of growth and 3.35 (SD = 0.78; *P* = 0.108) after 48 h of growth.

Different carbon sources were supplemented to the growth medium to further assess the acidifying compound in the S. aureus supernatant. The addition of glycerol resulted in supernatants with pH values around 4.8, similar to when glucose was added (Fig. 1D). Succinate, malic acid, and sodium pyruvate did not decrease the pH in the supernatant and were therefore discarded from further analysis (Fig. 1D).

These results suggested glucose metabolism is essential in the production of the P. aeruginosa inhibiting substance. In support of this, the viability of P. aeruginosa was only affected when cultured in supernatants with 1% glycerol and 1% glucose, and the low pH (4.8) itself did not affect the growth (Fig. 1E).

### The growth-inhibiting substance was glucose concentration-dependent and pH-dependent.

**FIG 2:**
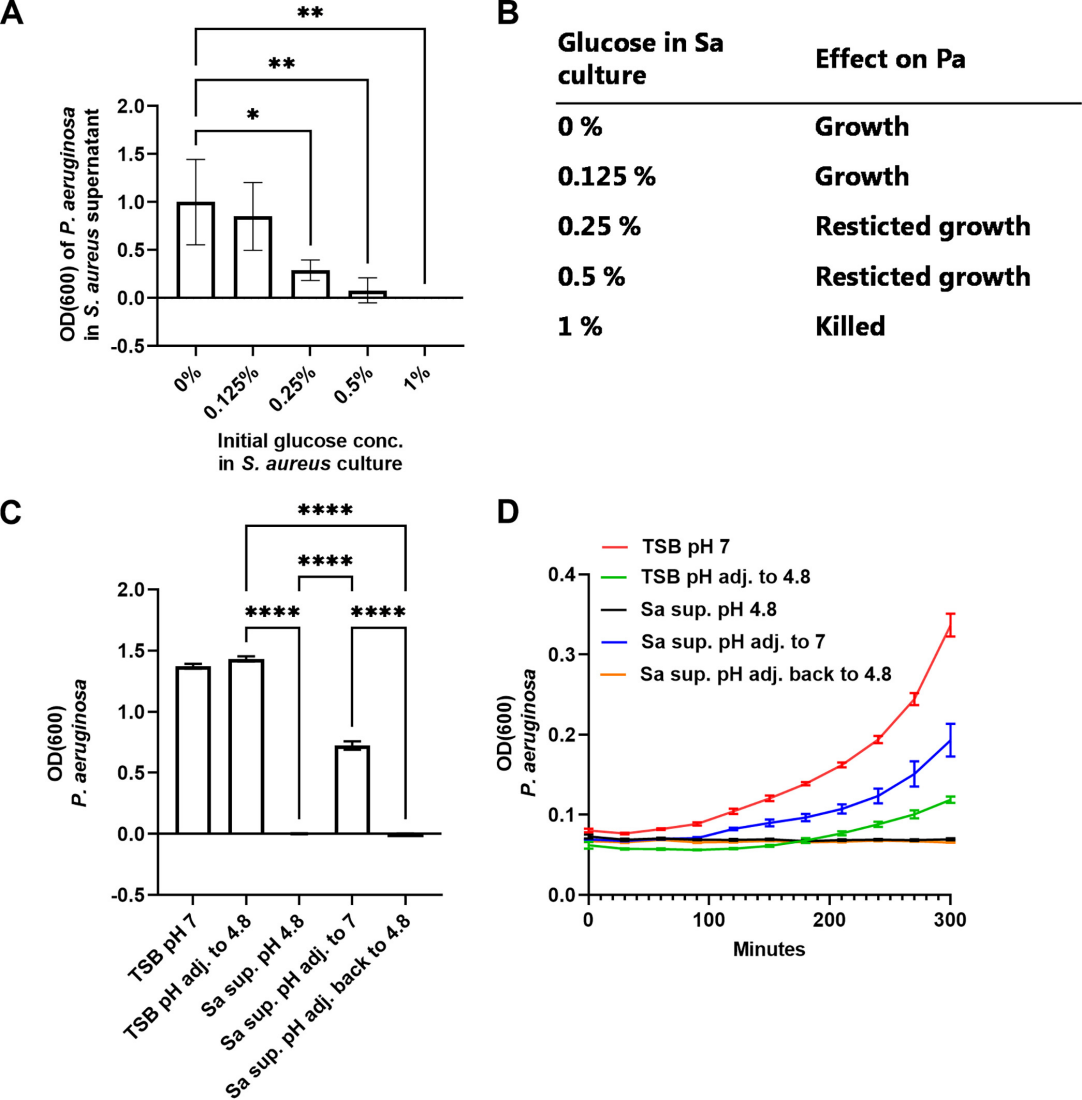
The growth-inhibiting substance was glucose concentration-dependent and pH-dependent. The relative growth (measured as absorbance at an optical density of 600 nm, OD_600_) of Pseudomonas aeruginosa in Staphylococcus aureus supernatants from overnight cultures with different glucose concentrations (A). Survival of P. aeruginosa in supernatants with 0 to 1% glucose (B). The relative growth (OD_600_) of P. aeruginosa when adjusting the pH of the same S. aureus supernatant up and down (C). The relative growth (OD_600_) of P. aeruginosa when adjusting the pH of the same S. aureus supernatant up and down within the first 5 h of growth (D). Error bars represent standard deviation (SD). Statistical difference was calculated using a one-way ANOVA with Dunnett correction (*n* = 3) (A and E) or Kruskal-Wallis with Dunn's correction (*n* = 3) (C). *, adjusted *P*  < 0.05; **, adjusted *P*  < 0.01; ****, adjusted *P* < 0.0001. Pa, P. aeruginosa; Sa, S. aureus; TSB, tryptic soy broth.

To determine at which glucose concentration the inhibiting substance was produced, P. aeruginosa was inoculated into S. aureus supernatants from overnight (ON) cultures with different glucose concentrations. The relative growth of P. aeruginosa was significantly inhibited in supernatants from ON cultures containing ≥ 0.25% glucose, while concentrations below 0.25% did not have an effect (Fig. 2A). Aliquots from these samples were subcultured to investigate at which glucose-concentration the inhibiting substance was bactericidal. P. aeruginosa survived in S. aureus supernatants from cultures containing 0% to 0.5% glucose and was killed in cultures containing 1% glucose (Fig. 2B).

The properties of the P. aeruginosa-inhibiting substance were further characterized in terms of pH. The relative growth of P. aeruginosa was restricted in S. aureus supernatants with 1% glucose with a pH = 4.8. However, when a subsample of the same supernatant was adjusted up to pH = 7, relative growth was observed. In line with this, adjusting the pH of the supernatant back to 4.8 resulted in restricted growth (Fig. 2C and D).

These results collectively indicated that the P. aeruginosa-inhibiting substance was glucose concentration-dependent and pH-dependent.

### The growth-inhibiting effect was concentration-dependent and affected the growth of other species.

**FIG 3:**
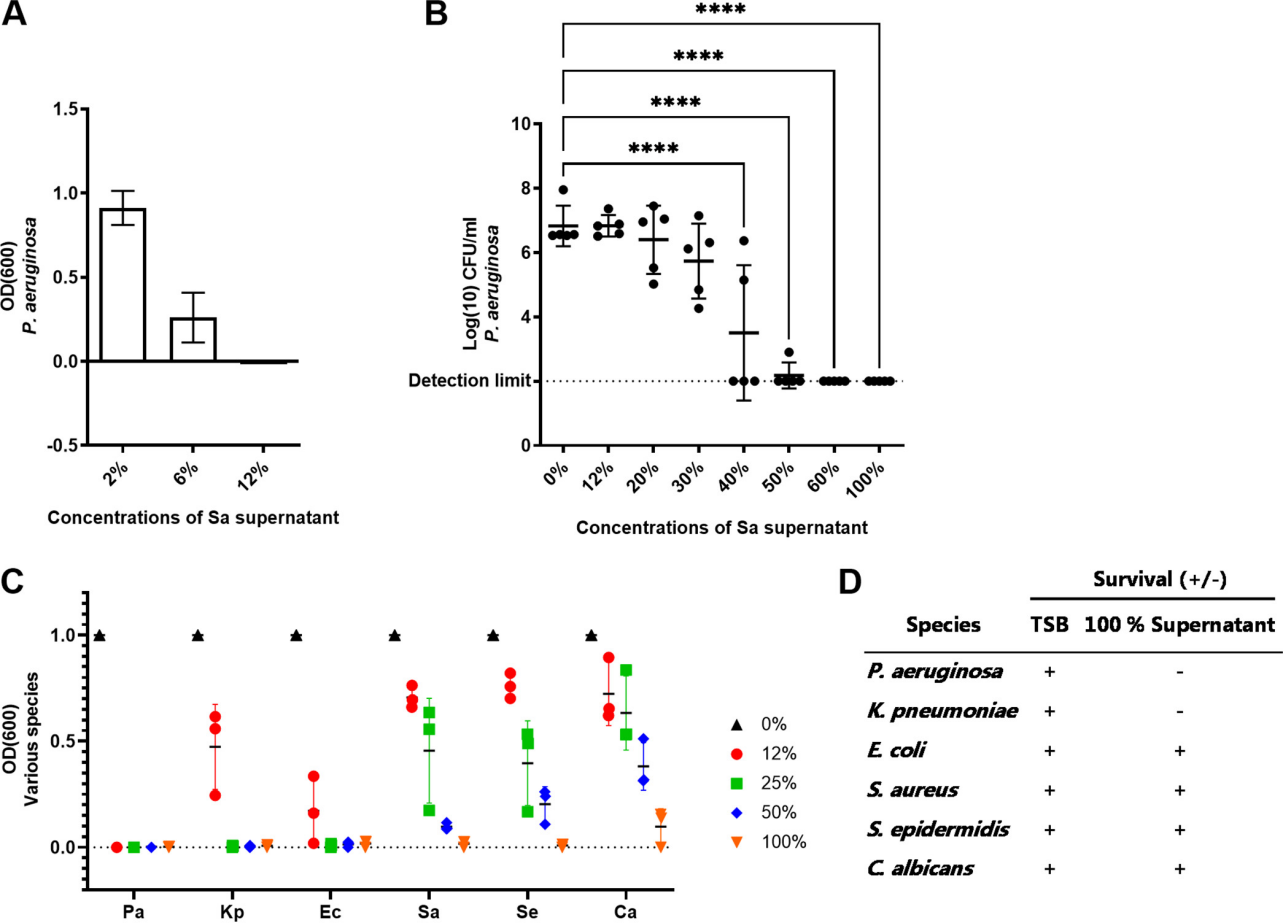
The growth-inhibiting effect was concentration-dependent and affected the growth of other species. The relative growth of Pseudomonas aeruginosa (measured as absorbance at an optical density of 600 nm, OD_600_) in diluted Staphylococcus aureus supernatants (A). Log-transformed CFU per milliliter (log_10_ CFU/mL) of P. aeruginosa in dilutions of S. aureus supernatants (B). Relative growth (OD_600_) of different species in dilutions of S. aureus supernatants (C). Survival (+/−) in S. aureus supernatant was determined by aliquots from overnight culturing (D). Error bars represent standard deviation (SD). Statistical difference was calculated using a one-way ANOVA with Dunnett correction (*n* = 5 (B), *n* = 3 (A and C)). ****, adjusted *P* < 0.0001. Pa, P. aeruginosa; Kp, K. pneumoniae; Ec, E. coli; Sa, S. aureus; Se, S. epidermidis; Ca, C. albicans.

When diluting the supernatant in tryptic soy broth (TSB), an effect on the growth of P. aeruginosa was observed in dilutions containing 6 and 12% supernatant (Fig. 3A). In contrast, the viability of P. aeruginosa was affected at ≥ 40% supernatant (*P* < 0.001), and the killing of P. aeruginosa was observed in TSB with ≥ 60% supernatant (Fig. 3B). More than 40% supernatant seemed to be the breakpoint as variation in growth was observed between the five replicates.

The growth-inhibiting effect of glucose-grown S. aureus supernatant was further tested on four other bacteria and one yeast species. While the growth of P. aeruginosa was restricted in 12% supernatant, the growth of two other Gram-negative species (Klebsiella pneumonia and Escherichia coli) was restricted in 25% supernatant (Fig. 3C). Nondiluted supernatant (100%) prevented the growth of all tested microorganisms, except for Candida albicans (Fig. 3C). Viability was assessed by plating aliquots of the microorganisms grown in 100% supernatant; P. aeruginosa and K. pneumoniae did not survive, while E. coli, S. aureus, S. epidermidis, and C. albicans survived under these conditions (Fig. 3D).

### S. aureus inhibited P. aeruginosa in a streak assay.

**FIG 4:**
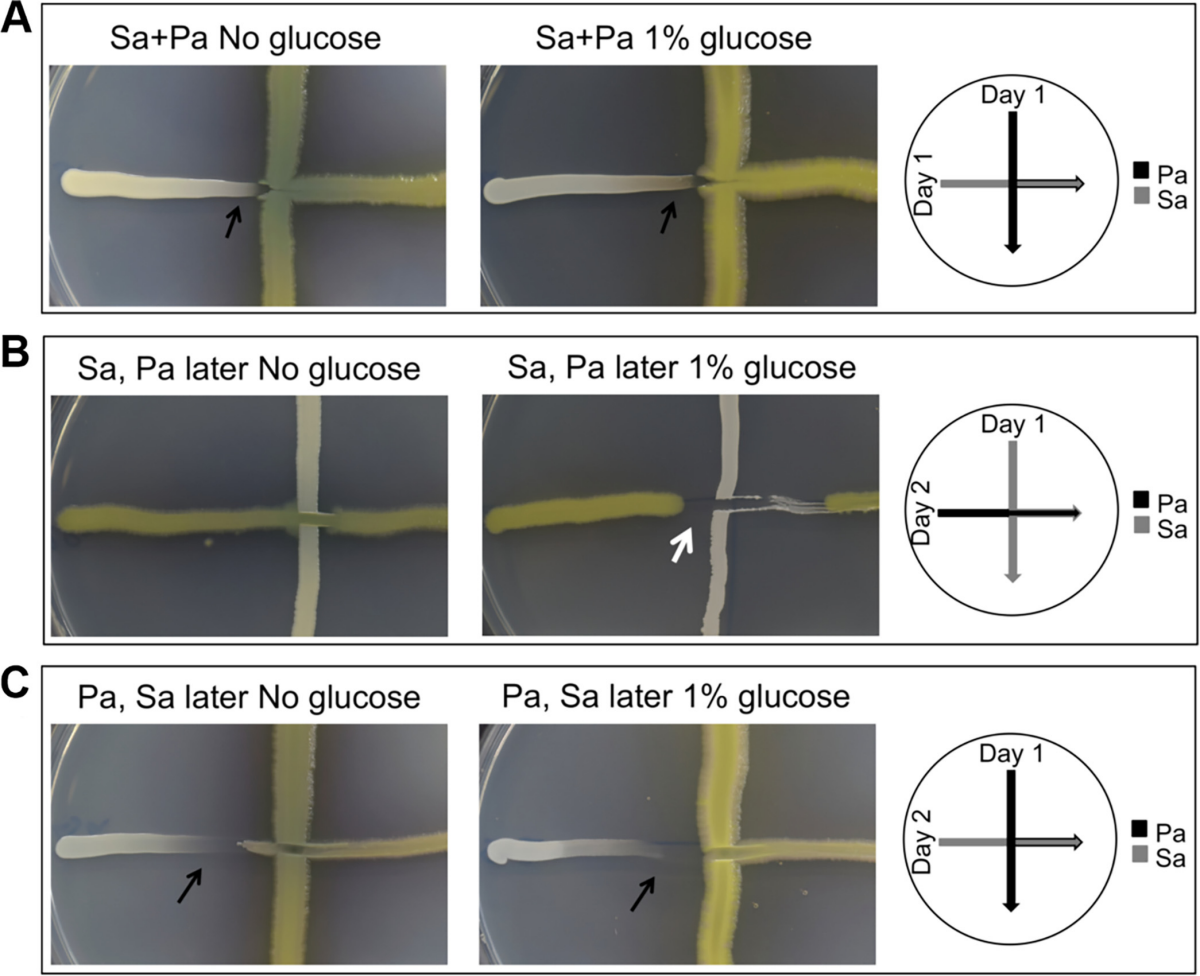
Staphylococcus aureus inhibits Pseudomonas aeruginosa in a streak-assay. Cross-streak assays with S. aureus and P. aeruginosa on LB agar plates +/− 1% glucose. Drawings to the right of the image show how bacteria were streaked. The white arrow indicates zones of P. aeruginosa inhibition, and the black arrows indicate zones of S. aureus inhibition (A to C). S. aureus and P. aeruginosa were inoculated together (Sa+Pa) or at 1-day intervals (Sa, Pa later).

Both strains were grown on culture plates to study how the two species interact when grown on plates. P. aeruginosa and S. aureus were cross-streaked on lysogenic broth (LB) agar plates +/− 1% glucose. When both species were streaked simultaneously, P. aeruginosa inhibited the growth of S. aureus in the presence and absence of glucose (Fig. 4A). When S. aureus was streaked on plates before P. aeruginosa, a P. aeruginosa-inhibiting effect was observed when glucose was present, while no inhibition was seen without glucose (Fig. 4B). When P. aeruginosa was streaked on plates before S. aureus, the growth of S. aureus was inhibited more than when the two species were streaked simultaneously (Fig. 4C).

### Identifying the inhibiting substance.

**FIG 5:**
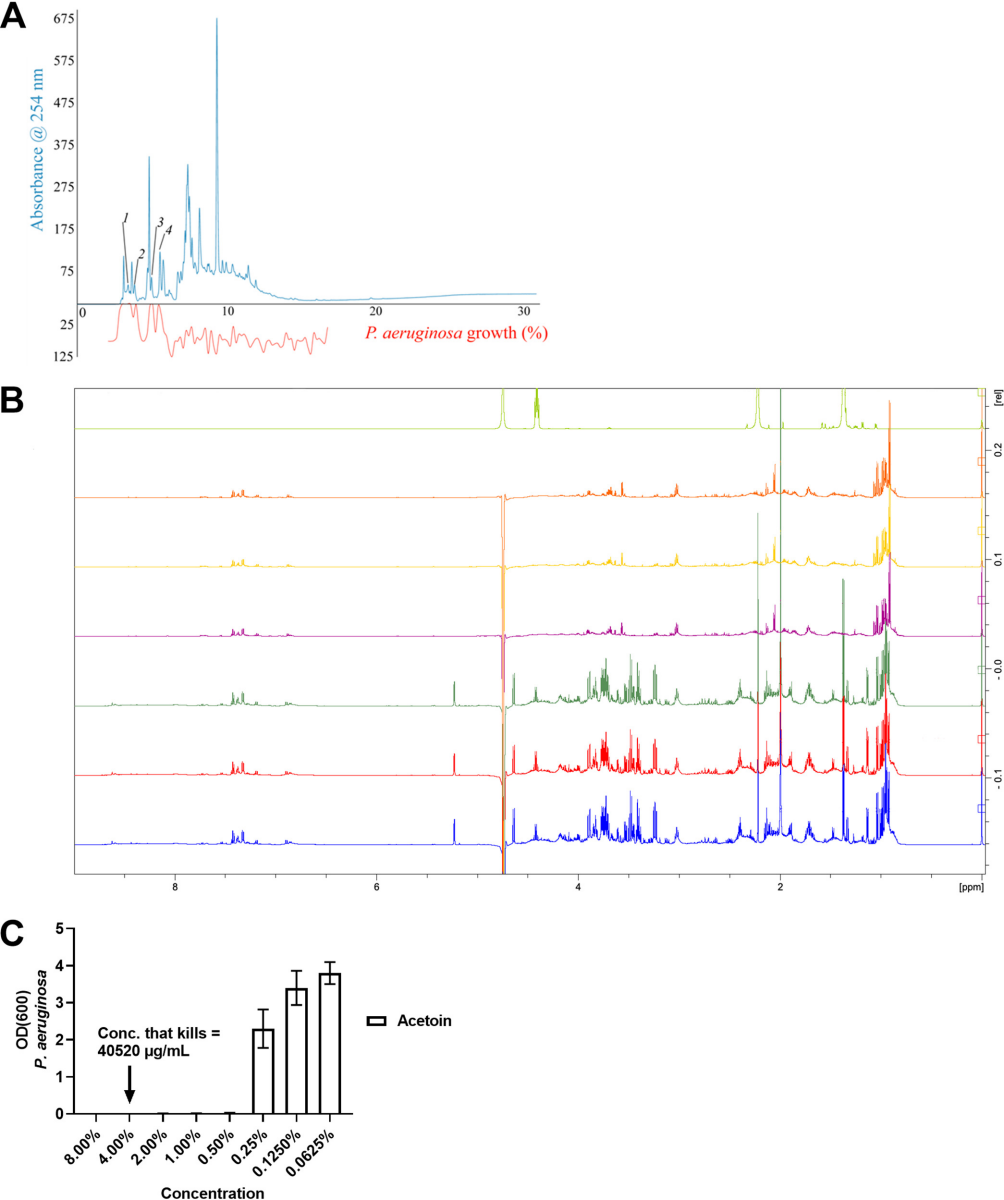
Identifying the inhibiting substance. HPLC chromatogram monitored at 254 nm (blue trace) and percentage inhibition of P. aeruginosa growth (red trace). HPLC peaks correlated with P. aeruginosa inhibition were observed in the retention time range of 2.5 to 6.0 min (A). ^1^H NMR spectra acquired at 600 MHz of S. aureus supernatants after triplicate cultivation with 1% glucose added to the growth medium (three spectra at the bottom) and after triplicate cultivation without glucose added to the growth medium (three spectra at the top) (B). The top spectrum is the acetoin reference spectrum. For both experiments *n* = 3. The relative growth (measured as absorbance at an optical density of 600 nm, OD_600_) of Pseudomonas aeruginosa in tryptic soy broth with different concentrations of acetoin (C). Error bars represent standard deviation (SD).

**(i) High-resolution P. aeruginosa inhibition profiling in S. aureus supernatant.** As a first approach to identifying the P. aeruginosa-inhibiting substance, the S. aureus supernatant was investigated with high-resolution P. aeruginosa inhibition profiling. Thus, an analytical-scale high-performance liquid chromatography (HPLC) method was developed to separate individual molecules in the supernatant and the eluate from 2.5 to 17.5 min. was fractionated into the wells of a 96-well microplate. Each fraction was subsequently inoculated with P. aeruginosa, and the growth inhibition of each well was calculated and plotted against each well's mean retention time from the fractionation. This resulted in a high-resolution P. aeruginosa inhibition profile (biochromatogram) with a resolution of 5.3 data points per minute plotted underneath the HPLC chromatogram, as shown in Fig. 5A. The principle of high-resolution inhibition profiling with other targets has been explained elsewhere (33, 34). The biochromatogram showed that the separated substances eluted with HPLC peaks in the retention time range of 2.5 to 6.0 min correlated with the P. aeruginosa inhibitory activity, whereas no P. aeruginosa inhibitory activity was observed with material eluted later than 6 min. Thus, it can be concluded that a series of very polar constituents caused the inhibitory activity. Several attempts were unsuccessful in developing an analytical-scale HPLC method with baseline separation of the individual constituents in the retention time range of 2.5 to 6.0 min. Consequently, it was impossible to construct a biochromatogram allowing correlation between fully separated constituents and P. aeruginosa inhibitory activity.

**(ii) The inhibiting substance was <3 kDa, and heat- and proteinase-stable.** Supernatant from S. aureus cultured with 1% glucose, filtered through a 3 kDa molecular weight cutoff filter, killed P. aeruginosa. This was not the case for the resuspended concentrate containing molecules >3-kDa (unpublished data). In addition, neither autoclaving nor proteinase treating the supernatant decreased the P. aeruginosa-inhibiting effect (unpublished data). Thus, the inhibiting substance was a heat- and proteinase-stable molecule with a molecular mass lower than 3 kDa. Characteristics of the P. aeruginosa-inhibiting substance are summarized in Table 1.

**TABLE 1:** Characteristics of the S. aureus-produced P. aeruginosa-inhibiting substance

Product of glycolysis
<3 kDa
Hydrophilic
Proteinase K resistant
Heat resistant
pH-dependent activity
Killed P. aeruginosa in higher concentrations and inhibited growth at lower concentrations
Inhibited G- bacteria more efficiently than G+ bacteria

**(iii) NMR chemical fingerprints of crude S. aureus supernatants.** To compare these chemical fingerprints, S. aureus supernatants (+/− 1% glucose) from ON cultures were subjected to 600 MHz ^1^H nuclear magnetic resonance (NMR) analysis (Fig. 5B). As expected, the supernatants from S. aureus with 1% glucose still contain glucose, as seen from the doublet signal of the anomeric proton of α-d-glucopyranoside (δ 5.19, d, *J*_H1'eq, H2'ax_ = 3.8 Hz) and β-d-glucopyranoside (δ 4.61, d, *J*_H1'ax, H2'ax_ = 7.9 Hz) and additional unresolved and/or overlapping signals in area δ3 to 4 ppm. However, there were also other differences between the two series of samples. Thus, a hump of broad unresolved signals in the range of 7.5 to 8.7 ppm, presumably amide N-H signals, suggested the presence of one or more oligopeptides or cyclic peptides.

In addition, the ^1^H NMR spectra of supernatant from S. aureus with 1% glucose (Fig. 5B bottom) showed a very intense singlet resonance signal (δ 1.96, s, CH_3_C=O) correlating with a signal at δ 24.9 in the heteronuclear single quantum coherence (HSQC) spectrum and at δ 182.1 in the heteronuclear multiple bond correlation (HMBC) spectrum. These signals agree with acetic acid compared to the Biological Magnetic Resonance Data Bank spectra.

In addition, intense signals were seen at δ 1.33 (3H, d, *J *= 7.2 Hz), δ 2.22 (3H, s), and δ 4.38 (1H, q, *J *= 7.2 Hz). These signals agreed with acetoin, as seen from the reference spectrum of acetoin inserted at the top of Fig. 5B.

The signals for acetic acid, amide N-H signals, and acetoin are not observed in the supernatants of S. aureus cultured without glucose. Likewise, the patterns for amino acid methyl groups in the region 0 to 9-1.1 ppm, i.e., for alanine, valine, isoleucine, and threonine, as well as the pattern for C_α_-H and other downfield signals in the region 3 to 4 ppm, were only seen in the spectra with glucose added, indicating an altered amino acid metabolism.

**(iv) Effect of acetoin.** Based on NMR results, acetoin was identified as one of the possible inhibitory compounds biosynthesized in S. aureus supernatants. To assess the bactericidal effect of acetoin, the relative growth of P. aeruginosa was tested at different concentrations. A concentration of 0.5% restricted the growth, and concentrations of 4% acetoin (40.520 μg/mL) killed P. aeruginosa (Fig. 5C).

## DISCUSSION

P. aeruginosa and S. aureus are highly prevalent in chronic wound infections and the lungs of patients with CF, and here they have been shown to coexist (10, 35, 36). When cocultured in experimental shaken cultures, P. aeruginosa quickly outcompetes S. aureus, and P. aeruginosa has been shown to inhibit S. aureus in various *in vitro* systems (35, 36). On the contrary, S. aureus has not been shown to influence the viability or growth of P. aeruginosa directly. Thus, there is a knowledge gap on how S. aureus and P. aeruginosa can coexist in infections. One suggestion is that P. aeruginosa induces the selection of the S. aureus small colony variant (SCV) phenotype in the lungs of people with CF. It is indicated that this phenotype is protected from P. aeruginosa and could contribute to the ability of the two species to coexist (18, 37). However, this phenomenon does not fully explain why P. aeruginosa does not outcompete S. aureus *in vivo* and why P. aeruginosa and S. aureus are found as separate aggregates in these infections (7, 11, 13). Therefore, we sought to investigate whether an established S. aureus population would influence the growth and survival of P. aeruginosa, and we hypothesized that glucose could play a role and could be metabolized by S. aureus, creating organic acids known to kill P. aeruginosa (26–28).

### Main findings.

S. aureus produced concentration-dependent and pH-dependent P. aeruginosa-inhibiting substances in the presence of glucose. The substances were heat- and proteinase-stable with a molecular mass lower than 3 kDa (Table 1). Further classification of the molecules was performed with high-resolution inhibition profiles, which have been successfully applied to identify bioactive constituents directly from crude extracts of natural products (38–41). The early elution of the active peaks suggests that the compounds identified in this study may present a high degree of hydrophilic groups that did not interact with the octadecylsilane groups from the stationary-phase (C_18_ column) and were, therefore, easily eluted by the aqueous mobile phase. Further analysis showed that a cocktail of glucose-mediated substances was created in S. aureus supernatants, including acetoin, acetic acid, and possibly oligopeptides or cyclic peptides. Their individual antibacterial properties are discussed below in more detail.

**(i) Acetoin.** A recent study has shown that trophic cooperation exists between S. aureus and P. aeruginosa, where S. aureus produces acetoin, which P. aeruginosa can metabolize, thereby attenuating cooperation between these two organisms (42). In addition, acetoin was measured from the sputum of CF patients, where S. aureus mono-infected patients displayed higher concentrations of acetoin than patients coinfected with P. aeruginosa and S. aureus. On average, around one mM. However, a few values were as high as 100 and even >1000 mM acetoin (42). We observed an inhibition of P. aeruginosa growth at 57.49 mM acetoin and killing at 459.59 mM (Fig. 5C). These findings supported our results in that S. aureus produced acetoin. However, we proposed that, depending on glucose availability and the resulting converted acetoin concentration, this equilibrium can be skewed to favor S. aureus survival. Differences exist between the two studies. Most notably, we added 1% glucose to the TSB medium, resulting in a concentration of approximately 55 mM, whereas initial concentrations were closer to 3 mM in their study. Thus, more acetoin could be produced in our setup. In addition, we cultured S. aureus for 18 to 24 h before separating the supernatant for further experimental testing, whereas they cultured S. aureus for 8 h.

**(ii) Acetic acid.** S. aureus is known to produce a variety of organic acids during glucose metabolism, including acetic acid, lactic acid, pyruvic acid, citric acid, succinic acid, and fumaric acid (43, 44). The antibacterial properties of weak organic acids have been recognized for generations and are well-documented (28, 45–47), more specifically an effect of acetic acid (28), lactic acid (48), and citric acid (49) has been shown for *P. aeruginosa*. The antimicrobial actions of organic acids are not fully understood. However, they are thought to involve membrane disruption and decreased intracellular pH. Like the S. aureus supernatant described in this study, organic acids can work both bacteriostatic and bactericidal (45, 46). Organic acids can penetrate the bacterial membrane in its uncharged form, and the lipophilicity of organic acids increases with decreasing pH. Therefore, the antibacterial effect of organic acids is pH-dependent (28, 45, 46). This phenomenon also applied to the P. aeruginosa-inhibiting effect of the S. aureus substances described in this study, as we observed a clear pH-dependent, anti-P. aeruginosa effect of the S. aureus supernatant (Fig. 2C). As shown before (28), it is not the low pH *per se* that causes the inhibition of P. aeruginosa, but the S. aureus-secreted substance because pH-adjusted medium with HCl did not inhibit P. aeruginosa (Fig. 1E). It has been shown that P. aeruginosa is more sensitive to acetic acid than S. aureus, S. epidermidis, and E. coli (50–52). In addition, C. albicans is less sensitive to acetic acid than P. aeruginosa (53). These findings agree with the results of our study.

**(iii) Peptides.** It has previously been shown that S. aureus produces bioactive peptides (54, 55), and the peptides observed in this study could arise from S. aureus*'s* biosynthetic pathway or through modification/degradation of casein-derived peptides present in the growth medium as previously described for *Lactobacillus* sp. cultures (56). However, this is speculative. Of particular interest, S. aureus harbors a type I toxin-antitoxin system that, when expressed, produces two peptides with activity against both G− and G+ positive bacteria, similar to what we have observed in our study (50).

### The microenvironment in infection sites.

Bacteria are notoriously known to produce different substances to combat the host immune system or other bacteria, thus increasing their chance of survival in harsh environments, and they must rely on what they can metabolize from their microenvironment. One such substance is glucose, and it is relevant to consider the glucose levels at infection sites.

Diabetes is a significant risk factor for developing chronic wounds, and elevated glucose levels in wounds of diabetic people are expected. Likewise, glucose can be detected in airway secretions in people with hyperglycemia (57, 58). CF-related diabetes is a common comorbidity and affects up to 50% of adults with CF (51). Thus, the glucose-mediated secretion of the P. aeruginosa-inhibiting substance could be expected to occur *in vivo*, suggesting these S. aureus-secreted substances to be clinically relevant. An effect on the relative growth of P. aeruginosa was observed at a concentration of 0.25% glucose, equal to 13.8 mmol/L, resembling the concentrations observed in the interstitial fluid of diabetic foot ulcers (8.0 ± 1 mmol/L) (52) and the sputum of patients with cystic fibrosis-related diabetes (range 0 to 64.4 mmol/L) (30). Furthermore, it has been shown that CF patients with high blood glucose levels are more likely to be co-infected with S. aureus and P. aeruginosa than patients with normal blood glucose levels (29), supporting the theory that glucose plays a role in facilitating the coexistence of P. aeruginosa and S. aureus.

We also found that the P. aeruginosa-inhibiting substances were active at lower pH values, i.e., P. aeruginosa growth inhibition was observed when pH in the supernatant was low (pH = 4.8) (Fig. 2). Airways of CF patients can be acidic; pH values of 3 to 6.5 have been reported, with decreased pH levels during exacerbations (59–61). The S. aureus-secreted P. aeruginosa-inhibiting substances would be active within this pH range. During normal healing of acute wounds, pH follows a course of being acidic, then alkaline, and as the healing progresses, the wound milieu becomes acidic again. On the contrary, chronic wounds are kept in an alkaline state, with pH secretions >7.3 (62). Under these conditions, the S. aureus-produced substances would not be expected to be active. However, local pH measurements in chronic wounds have shown that pH varies between and within wounds, including acidic areas (63). Therefore, it could be speculated that the P. aeruginosa-inhibiting substances will be active in some areas of chronic wounds where pH is low or that other factors may be necessary for S. aureus and P. aeruginosa to coexist in chronic wounds.

**FIG 6:**
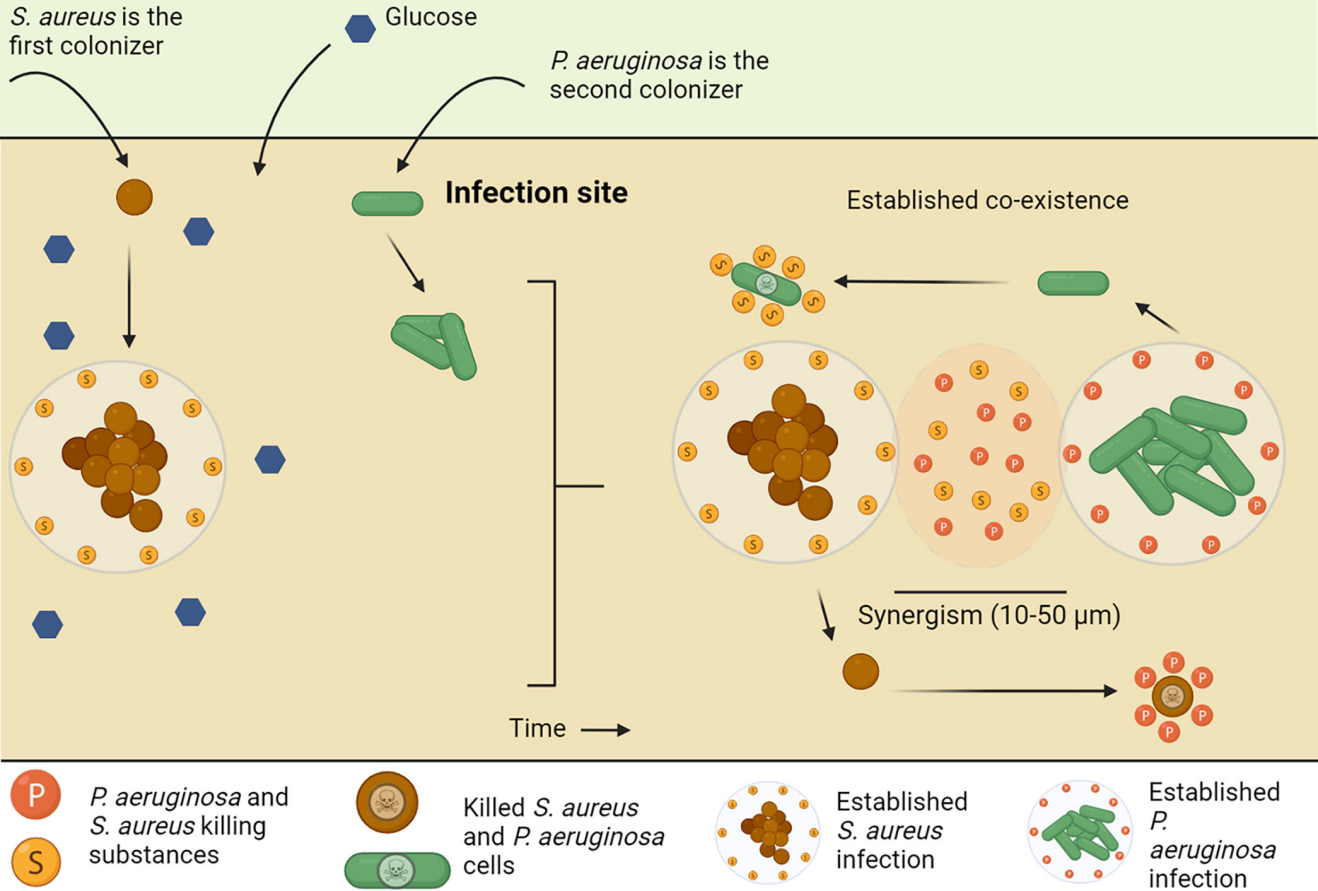
The S. aureus head-start hypothesis. S. aureus colonizes the infection site before P. aeruginosa and establishes a population that produces the P. aeruginosa-inhibiting substance when glucose is present. P. aeruginosa colonizes later and is prevented from mixing and overgrowing S. aureus. P. aeruginosa establishes populations within a distance to S. aureus (calling-distance,10 to 50 μm, refer to reference (68)), and P. aeruginosa also produces S. aureus-inhibiting substances keeping the two species apart. Thereby, the coexistence of the two species is seen as separated single-species aggregates in coinfections, with synergy as a result, e.g., as increased virulence and antibiotic tolerance.

Thus, we have shown that S. aureus can secrete a cocktail of P. aeruginosa-inhibiting substances, and we hypothesize that the conditions in the lungs and chronic wounds support the production and activity of these substances, preventing S. aureus from being eradicated by P. aeruginosa *in vivo* and contributing to the coexistence of these two species in chronic infections. S. aureus colonizes wounds and lungs before P. aeruginosa colonization (6, 64), and S. aureus is assumed to have established a population when encountering P. aeruginosa. We hypothesize that such established S. aureus populations can secrete the P. aeruginosa-inhibiting substances, thereby giving S. aureus a “head start,” preventing S. aureus from being overgrown by P. aeruginosa. This hypothesis is schematically summarized in Fig. 6.

Lastly, it must be mentioned that other factors might influence the coexistence of these two species where factors such as the timing of inoculation, model system (biofilm versus planktonic), and temperature can affect the co-existence (65, 66), adding to the complexity of this interspecies relationship. In addition, these findings explain the co-existence of these two bacteria in chronic infections; however, the authors cannot exclude that this could be true for some acute infections such as respiratory infections where S. aureus has had a “head start.”

With our finding of S. aureus-produced, P. aeruginosa-inhibiting substances, we provided new insights into how these two bacterial species can co-exist in infections and thereby improve understanding of this complex interaction. We hope that identifying these S. aureus-produced substances could let us explore the possible future usage in antibacterial treatment strategies. In addition, these results might be used to facilitate the co-existence of these two bacteria in *in vitro* models for further testing of their complex relationship.

## MATERIALS AND METHODS

### Bacterial strains and growth conditions.

P. aeruginosa, PAO1, obtained from the Pseudomonas Genetic Stock Center (PGSC) (https://www.pseudomonas.med.ecu.edu), and S. aureus 8325-4 (67), were used throughout the study when nothing else is indicated. Other bacteria used in this study are listed in Table S1 in Supplemental File 1. Clinical S. aureus and P. aeruginosa strains were pairwise isolated from the same CF patients, referred to as co-isolated.

Overnight (ON) cultures were prepared from frozen stocks and incubated for 18 to 24 h at 37°C with a shake at 180 rpm. Tryptic soy broth (TSB) (BD Diagnostics, Heidelberg, DE) was used to grow S. aureus. TSB was supplemented with 0 to 1% glucose (Panum Institute Substrate Department, University of Copenhagen, Copenhagen, DK) as described in the respective sections. Lysogeny broth (LB) was used for P. aeruginosa and other organisms. LB (pH 7.5) consisted of five g/liter yeast extract (Oxoid, Roskilde, DK), 10 g/liter tryptone (Oxoid), and 10 g/liter NaCl (Merck, NYC, USA). LB broth and LB agar plates were supplied by the Panum Institute Substrate Department.

### Cell-free S. aureus supernatants.

All cell-free supernatants were prepared from ON cultures by spinning them for 10 min at 3500 × *g* and then sterile filtering the supernatant through a 22 μm filter (TPP, Trasadingen, CH).

### Glucose tolerability and restriction of P. aeruginosa growth.

ON cultures of S. aureus were diluted to an optical density at 600 nm (OD_600_) of 0.01 in TSB with increasing glucose concentrations (0 to 1%), shaking at 180 rpm at 37°C. Relative growth, determined by optical density at 600 nm, OD_600_ (UV-1800 UV-VIS spectrophotometer, Shimadzu, Japan), and pH (827 pH lab, Metrohm, CH) were determined after ON culturing.

A similar approach was conducted with various S. aureus strains (see Table S1 in Supplemental File 1), where pH was measured in ON cultures grown with 1% glucose. In addition, supernatants were prepared from ON cultures, and P. aeruginosa was inoculated into supernatant with an optical density of 0.01 (OD_600_). Culture flasks were cultured ON at 37°C, shaking at 180 rpm. The viability of P. aeruginosa was assessed by plating aliquots from the ON cultures on LB agar plates (growth or no growth). Plates were incubated ON at 37°C.

### S. aureus and P. aeruginosa coculture.

ON cultures of S. aureus and P. aeruginosa were reinoculated into fresh TSB and grown for 3 to 4 h before being cocultured 1:1 (OD_600_ = 0.1) in TSB +/− 1% glucose in culture flasks, shaking at 180 rpm at 37°C. Aliquots of the cultures were sampled over time, serially diluted, and plated on Pseudomonas isolation agar (PIA) (Sigma-Aldrich, St. Louis, MO, USA) and 7.5% sodium chloride agar (Panum Institute Substrate Department) to determine the viability (CFU per milliliter, CFU/mL) of P. aeruginosa and S. aureus, respectively. Plates were incubated ON at 37°C before counting colonies.

### Carbon sources supported S. aureus growth and lowered the pH in the supernatant.

S. aureus shaking cultures (OD_600_ = 0.01 in TSB) with different carbon sources: glycerol, succinate, malic acid, and sodium pyruvate (Sigma-Aldrich), were prepared with equal concentrations of C-atoms. Start pH in the culture was adjusted with hydrochloric acid (HCl) or sodium hydroxide (NaOH) to 7.3 before incubation, and pH was measured after 24 h of growth.

### Supernatant with different carbon sources.

P. aeruginosa viability was tested in S. aureus supernatants grown with carbon sources that lowered pH (glucose and glycerol). P. aeruginosa was diluted to OD_600_ = 0.01, inoculated into the supernatant, and incubated ON at 37°C with shaking (180 rpm). Viability was determined by subsequent CFU/mL at different time points, and plates were incubated ON at 37°C before counting colonies.

### P. aeruginosa growth in S. aureus supernatant.

All supernatant samples described below started with P. aeruginosa being inoculated into the S. aureus supernatant (start OD_600_ = 0.01) and incubated ON at 37°C with shaking (180 rpm). The relative growth of P. aeruginosa was determined by measuring OD_600_, and viability was determined either by CFU/mL or spotting aliquots on LB agar plates (growth or no growth). Plates were incubated ON at 37°C. pH was adjusted with either HCl or NaOH in all the experiments.

### Glucose concentration.

Supernatants from S. aureus cultures grown with increasing glucose concentrations (0 to 1%) were prepared to test the effect of glucose on the subsequent relative growth (OD_600_) of P. aeruginosa. All supernatants were pH-adjusted to pH 4.8 before inoculation. In addition, aliquots from ON cultures samples were spotted on LB agar plates to determine the viability of P. aeruginosa (growth or no growth). Plates were incubated ON at 37°C.

### Supernatant pH.

Supernatants from S. aureus grown with 1% glucose were adjusted to pH values between 4.8 and 7.0 to determine the effect of pH on the relative growth (OD_600_) of P. aeruginosa. Fresh TSB was also adjusted to pH values 4.8 and 7.0 as controls. In addition, aliquots from ON cultures were spotted on LB agar plates to determine the viability of P. aeruginosa (growth or no growth). Plates were incubated ON at 37°C.

### Supernatant concentrations.

Supernatant from S. aureus grown with 1% glucose was diluted in fresh TSB and adjusted to pH 4.8 to determine at which concentration the supernatant affected the relative growth (OD_600_) of P. aeruginosa. Based on these results, 12% supernatant was used as the lowest tested concentration in the subsequent viability experiments.

The viability of P. aeruginosa, Klebsiella pneumoniae, Escherichia coli, S. aureus, Staphylococcus epidermidis, and Candida albicans (see Table S1 in Supplemental File 1) was assessed in diluted S. aureus supernatant. All strains started with an OD_600_ = 0.01. For P. aeruginosa, viability was determined by CFU/mL, while aliquots were spotted on LB agar plates for the remaining strains (growth or no growth). Plates were incubated ON at 37°C.

### S. aureus and P. aeruginosa streak assay.

P. aeruginosa and S. aureus from ON cultures were cross streaked on LB agar plates supplemented with 1% glucose or LB agar plates without glucose. Initially, one strain was streaked out, and then, when the plate was dry, the other strain was cross-streaked. The two strains were streaked on the same day or at 1-day intervals. Plates were incubated at 37°C for 2 days and then inspected visually.

### High-resolution P. aeruginosa inhibition profiling.

Microfractionation of supernatant from S. aureus supernatants grown with 1% glucose was performed using an Agilent 1200 system comprising a G1311A quaternary pump, a G1322A degasser, a G1316A thermostatted column compartment, a G1315C photodiode-array detector, a G1367C high-performance autosampler, and a G1364C fraction collector, controlled by Agilent ChemStation version B.03.02 software (Agilent, Santa Clara, CA, USA). Separations were performed using a reversed-phase Phenomenex C_18_(2) Luna column (150 mm × 4.6 mm i.d., 3 μm particle size, 100 Å pore size) (Phenomenex, Torrance, CA, USA). A binary elution gradient of water:acetonitrile (95:5, solvent A) and acetonitrile:water (95:5, solvent B), both acidified with 0.1% formic acid, was used for the following elution gradient: 0 min, 0% B; 5 min, 8% B; 40 min, 20% B; 75 min, 60% B; 76 min, 100% B; 80 min, 100% B, under a flow rate of 0.5 mL/min. The chromatographic eluate was collected into 96-well microplates from 2 to 17 min in 80 wells. The fractions were subsequently evaporated to dryness using a Savant SPD121P speed vacuum concentrator coupled with an RVT400 refrigerated vapor trap and an OFP-400 oil-free pump. Fractions were redissolved in fresh TSB and adjusted to pH 4.8. The relative growth (OD_600_) of P. aeruginosa was tested in each fraction and plotted at the mean retention time of the corresponding well. Plates were incubated ON at 37°C.

### HPLC-PDA-HRMS analysis of S. aureus supernatant.

High-performance liquid chromatography, photodiode array detection, high-resolution mass spectrometry (HPLC-PDA-HRMS) experiments of S. aureus supernatant with and without 1% glucose were performed on an Agilent 1260 HPLC system (Agilent, Santa Clara, CA, USA) consisting of a G1311B quaternary pump with a built-in degasser, a G1329B autosampler, a G1316A thermostatted column compartment, and a G1315D photodiode array detector. A 20 μL aliquots of the sample was injected using the same conditions, column, and solvent system as described above, and a T-piece after the HPLC column directed approximately 1% of the eluate to a Bruker micrOTOF-Q II mass spectrometer equipped with electrospray ionization (ESI) source (Bruker Daltonik, Bremen, DE). Spectra were acquired in positive ionization mode, using a drying temperature of 200°C, a capillary voltage of 4100 V, a nebulizer pressure of 2.0 bar, and a drying gas flow of 7 L/min. A solution of sodium formate clusters was automatically injected at the beginning of the run to enable internal mass calibration. The remaining 99% of the eluate was directed to the photodiode array detector and monitored at a wavelength of 254 nm.

### NMR experiments.

NMR spectra were recorded in deuterated water, or methanol-*d*_4_ on a Bruker Avance III system (^1^H operating frequency of 600.13 MHz, ^13^C 150.90 MHz) equipped with a Bruker SampleJet sample changer and a 1.7-mm cryogenically cooled gradient inverse triple-resonance TCI probe-head (Bruker Biospin, Rheinstetten, DE) at 300 K. The ^1^H and ^13^C chemical shifts were referenced to the residual solvent signal of methanol-*d*_4_ at δ_H_ 3.31 ppm and δ_C_ 49.00 ppm, respectively. ^1^H NMR spectra were recorded using 30° pulses with a spectral width of 20 ppm, the acquisition time of 2.72 s, relaxation delay of 1.0 s, and 64k data points. Phase-sensitive double-quantum filtered correlation spectroscopy (DQF-COSY) and rotating-frame overhauser effect spectroscopy (ROESY) spectra were recorded using a gradient-based pulse sequence with 12 ppm spectral width and 2k × 512 data points (processed with forwarding linear prediction to 1k data points). HSQC spectra were recorded with 12 ppm spectral width for ^1^H and 200 ppm for ^13^C, 2k × 256 data points (processed with forwarding linear prediction to 1k data points), and a relaxation delay of 1.0 s. HMBC spectra were recorded with 12 ppm spectral width for ^1^H and 240 ppm for ^13^C, 2k × 128 data points (processed with forwarding linear prediction to 1k data points), and relaxation delay of 1.0 s. Icon NMR, ver. 4.2 (Bruker Biospin) was used to control automated NMR data acquisition, and NMR data were processed using Topspin, ver. 4.0.6 (Bruker Biospin).

### Supernatant fractions.

Supernatant from S. aureus grown in TSB + 1% glucose was size fractioned using 3-kDa molecular weight cutoff filters according to the manufacturer's protocol (Merck) to determine how fractions affected P. aeruginosa growth. P. aeruginosa was inoculated into the supernatant filtrate and incubated ON. In addition, P. aeruginosa was also inoculated into the concentrate from the filter resuspended medium (TSB, pH = 4.8) and incubated ON.

The same procedure was repeated where the filtrate was treated with proteinase K or autoclaved before incubation to see if it affected the P. aeruginosa inhibiting substance. Viability was determined by aliquots spotted on LB agar plates (growth or no growth). Plates were incubated ON at 37°C.

### Effect of acetoin.

Based on NMR results, acetoin was the main component that differentiated between the S. aureus supernatant supplemented with and without 1% glucose. The effect of acetoin was therefore tested on P. aeruginosa growth. Serial dilutions of acetoin were performed in pH-adjusted (pH = 4.8) TSB in cell-culture tubes (Thermo Fisher Scientific, Altrincham, UK). P. aeruginosa ON cultures were adjusted to OD_600_ 0.01, inoculated into pH-adjusted TSB with decreasing concentrations of acetoin (Sigma-Aldrich), and incubated for 18 to 24 h at 37°C. The relative growth of P. aeruginosa was determined by optical density (OD_600_), and viability was determined by spotting aliquots from liquid cultures on LB plates (growth or no growth). Plates were incubated ON at 37°C.

### Statistical analysis.

Unless otherwise stated, all experiments were performed in a minimum of 3 biological replicates. CFU/mL was log-transformed to ensure normally distributed data. Data were analyzed with GraphPad Prism 9.3.1 (GraphPad Software, La Jolla, CA, USA) using a one-way or two-way analysis of variance (ANOVA). A Geisser-Greenhouse correction was used for two-way ANOVA with Tukey's test to correct for multiple comparisons. A Dunnett's test was used to correct for multiple comparisons when using one-way ANOVA. A Kruskal-Wallis test was used for data sets that did not fulfill a Gaussian distribution, and Dunn's test was used to correct for multiple comparisons. *P < *0.05 was considered significant.

### Data availability.

The data sets generated during and/or analyzed during the current study are available from the corresponding author upon request.
