# Supplementary material for: Investigation of the Mechanism and Chemistry Underlying Staphylococcus aureus' Ability to Inhibit Pseudomonas aeruginosa Growth In Vitro
Source: J Bacteriol. 2022 Oct 11;204(11):e00174-22. doi: 10.1128/jb.00174-22 (PMC9664952; doi:10.1128/jb.00174-22)
Supplement: Supplemental file 1 — Table S1. Download jb.00174-22-s0001.pdf, PDF file, 0.4 MB [file jb.00174-22-s0001.pdf]

SUPPLEMENTAL MATERIAL

Supplemental Table S1

Strain	Description	Reference
Pseudomonas aeruginosa		
PAO1	Laboratory strain	PGSC
5x lung isolates	Clinical CF lung isolates	This study
Staphylococcus aureus		
8325-4	Laboratory strain	(68)
RN4220	Laboratory strain	(69)
MRSA-M2	Clinical osteomyelitis isolate	(70)
SA564	Human isolate	(71)
5x lung isolates	Clinical CF lung isolates	This study
USA300 JE2 wt	Community-associated MRSA strain	(72)
Klebsiella pneumonia	Clinical lung isolate	This study
Escherichia coli DH5α	Cloning strain	Life Technologies
Staphylococcus epidermidis	Clinical isolate	This study
Candida albicans SC5314	Wild type laboratory strain	(73)

Table S1 - Bacterial strains used in this study. PGSC = Pseudomonas Genetic Stock Center (http://www.pseudomonas.med.ecu.edu)
